# Age Alters Chromatin Structure and Expression of SUMO Proteins under Stress Conditions in Human Adipose-Derived Stem Cells

**DOI:** 10.1038/s41598-018-29775-y

**Published:** 2018-07-31

**Authors:** Xiaoyin Shan, Cleresa Roberts, Yemin Lan, Ivona Percec

**Affiliations:** 1 Department of Surgery, University of Pennsylvania, Philadelphia, PA 19104 USA; 2 Epigenetics Institute, Department of Cell and Developmental Biology, University of Pennsylvania, Philadelphia, PA 19104 USA

## Abstract

Adult stem cells play a critical role in tissue homeostasis and repair. Aging leads to a decline in stem cells’ regenerative capacity that contributes significantly to the maintenance of organ and tissue functions. Age-dependent genomic and epigenetic modifications together play a role in the disruption of critical cellular pathways. However, the epigenetic mechanisms responsible for the decline of adult stem cell functions remain to be well established. Here, we investigated age-dependent, genome-wide alterations in the chromatin accessibility of primary human adipose-derived stem cells (ASCs) in comparison to age-matched fibroblasts via ATAC-seq technology. Our results demonstrate that aging ASCs possess globally more stable chromatin accessibility profiles as compared to aging fibroblasts, suggesting that robust regulatory mechanisms maintain adult stem cell chromatin structure against aging. Furthermore, we observed age-dependent subtle changes in promoter nucleosome positioning in selective pathways during aging, concurrent with altered small ubiquitin-related modifier (SUMO) protein expression under stress conditions. Together, our data suggest a significant role for nucleosome positioning in sumoylation pathway regulation in stress response during adult stem cell aging. The differences described here between the chromatin structure of human ASCs and fibroblasts will further elucidate the mechanisms regulating gene expression during aging in both stem cells and differentiated cells.

## Introduction

Aging is characterized by a progressive decline in intrinsic tissue physiology and homeostasis. Adult stem cells are crucial for the regeneration of functionally differentiated cells in the maintenance of organismal homeostasis. However, stem cells are themselves subject to the aging process through the accumulation of toxic metabolites, DNA damage, epigenetic alterations, aggregation of damaged proteins, and mitochondrial dysfunction^1^. Additionally, exhaustion of the stem cell pool through impaired intrinsic regenerative capacity further contributes to the aging process^1^. Age-related genomic and epigenetic changes both influence cellular pathways during adult stem cell aging.

Primary human adipose-derived stem cells (ASCs) provide a robust model system for studying stem cell aging due to their relative abundance and accessibility. ASCs have been used to study age-associated decline of differentiation and regeneration potentials, among other pathways^2–6^, and their application in clinical regenerative medicine has similarly been extensively explored^7^. However, despite these investigations, our understanding of human ASC aging, remains to be well elucidated. We previously examined the transcriptome of ASCs and terminally differentiated fibroblasts during aging^8^ and demonstrated that in contrast to fibroblasts, ASCs maintain globally stable transcriptomes during aging. Several specific pathways, however, demonstrated age-dependent differential gene expression during aging in a cell-specific fashion. For example, genes involved in cell cycle control were up-regulated in aging ASCs but not in aging fibroblasts.

It has been well documented that the regulation of transcription involves numerous factors and cascading pathways that lead to specific interactions of regulatory factors with DNA binding motifs in genomic control regions such as promoters^9^. In eukaryotes, the chromatin structure regulation of transcription factor binding accessibility represents a significant level of control for modulating gene expression^9,10^. Age-related alterations in the chromatin structure have been observed in both yeast and mammals^11^. For example, in yeast, *Saccharomyces cerevisiae* (*S*. *Cerevisiae)*, global transcriptional up-regulation was associated with nucleosome loss during aging^12^. Similarly, mouse germinal vesicle stage oocytes demonstrate changes in histone methylation during aging^13,14^ and aging human CD8 T cells are characterized by changes in chromatin openness patterns^15^.

Nucleosomes are comprised of a 146 bp DNA sequence coiled around 8 histone protein cores and represent the fundamental units of eukaryotic chromatin. Specific alterations in nucleosome positioning in promoter regions are associated with gene expression regulation^16^. In general, nucleosome remodeling in response to physiological perturbations, including the eviction, appearance, or repositioning of one or two nucleosomes in the promoter region, typically increases the accessibility of binding sites for transcription factors that mediate transcriptional changes^17^. However, nucleosome positioning is not an absolute determining factor for gene transcription activity and likewise, major shifts in gene expression are not always associated with changes in nucleosome configuration^18^. For example, in *S*. *Cerevisiae*, the nucleosome position permits the binding of transcription factors Rap1 and Fhl1 to ribosomal gene promoters in the absence of active transcription^17^. It is clear that the effect of nucleosome position on gene transcription is complex^19^ and that predicting transcriptional activity based on promoter nucleosome position is not always directly feasible^17^, as transcription activation requires other regulators. The presence of these factors could be dynamic, such as when responding to environmental stimuli. A conserved feature of aging across organisms is the induction of stress response pathways in which signaling molecules and transcription regulators direct adaptive responses to various forms of cellular damage^20^. However, the stress response and repair mechanisms themselves become impaired during aging, as observed in *Caenorhabditis elegans* (*C*. *elegans*), where aging results in decreased resistance to multiple stresses and dysfunctional stress response pathways^21^. Although the regulatory molecules involved in the heat shock response (HSR) are known to become impaired during aging^22^, the regulation of HSR in human adult stem cells and the precise molecular mechanisms governing aging in these cells remain poorly understood. Importantly, the relationship between nucleosome position and transcriptional activity during aging has yet to be studied in primary human stem cells. As such, the elucidation of this mechanism is crucial to advancing our knowledge of transcriptional regulation in primary human cells.

Following our investigation of the age-dependent changes in trancriptome profiles of ASCs, we examined the epigenetic contribution of chromatin accessibility to gene expression regulation during aging of stem cells as compared to differentiated cells in this study. We employed ATAC-seq technology to investigate the genome-wide chromatin accessibility profiles of ASCs and fibroblasts in an age-dependent manner. We observed that the global chromatin structure of ASCs is more accessible than that of fibroblasts. Furthermore, the position of nucleosomes flanking TSSs is well maintained globally during aging in ASCs, but not in fibroblasts, suggesting that a robust regulatory mechanism supports a stable chromatin structure in stem cells as compared to somatic cells during aging.

Despite the age-dependent differences in chromatin stability between ASCs and fibroblasts, we observed subtle differential changes in nucleosome positioning in the promoters of genes encoding SUMO proteins. Stress response pathways are regulated at multiple levels, including by sumoylation, a post-translational protein modification by small ubiquitin-related modifier (SUMO)^23–25^. Alterations in global protein sumoylation have been observed in cultured human cells after heat shock, an acute proteotoxic stressor^26,27^. Although the difference in the promoter nucleosome position did not affect SUMO protein expression of the two cell types under non-stressed conditions, a significant change in SUMO-1 protein expression was observed in old ASCs under stress conditions, representing increased sensitivity to stress-regulated SUMO-1 protein expression in old as compared to young ASCs, an age-related change that was not observed in old fibroblasts when compared to young fibroblasts. The data presented here suggest a mechanism in which age-dependent alterations in the transcriptional activity of sumoylation factors are regulated by shifts in chromatin accessibility via nucleosome re-positioning.

## Results

### The chromatin accessibility profiles of ASCs and fibroblasts are distinctively different at baseline and during aging

**Figure 1:**
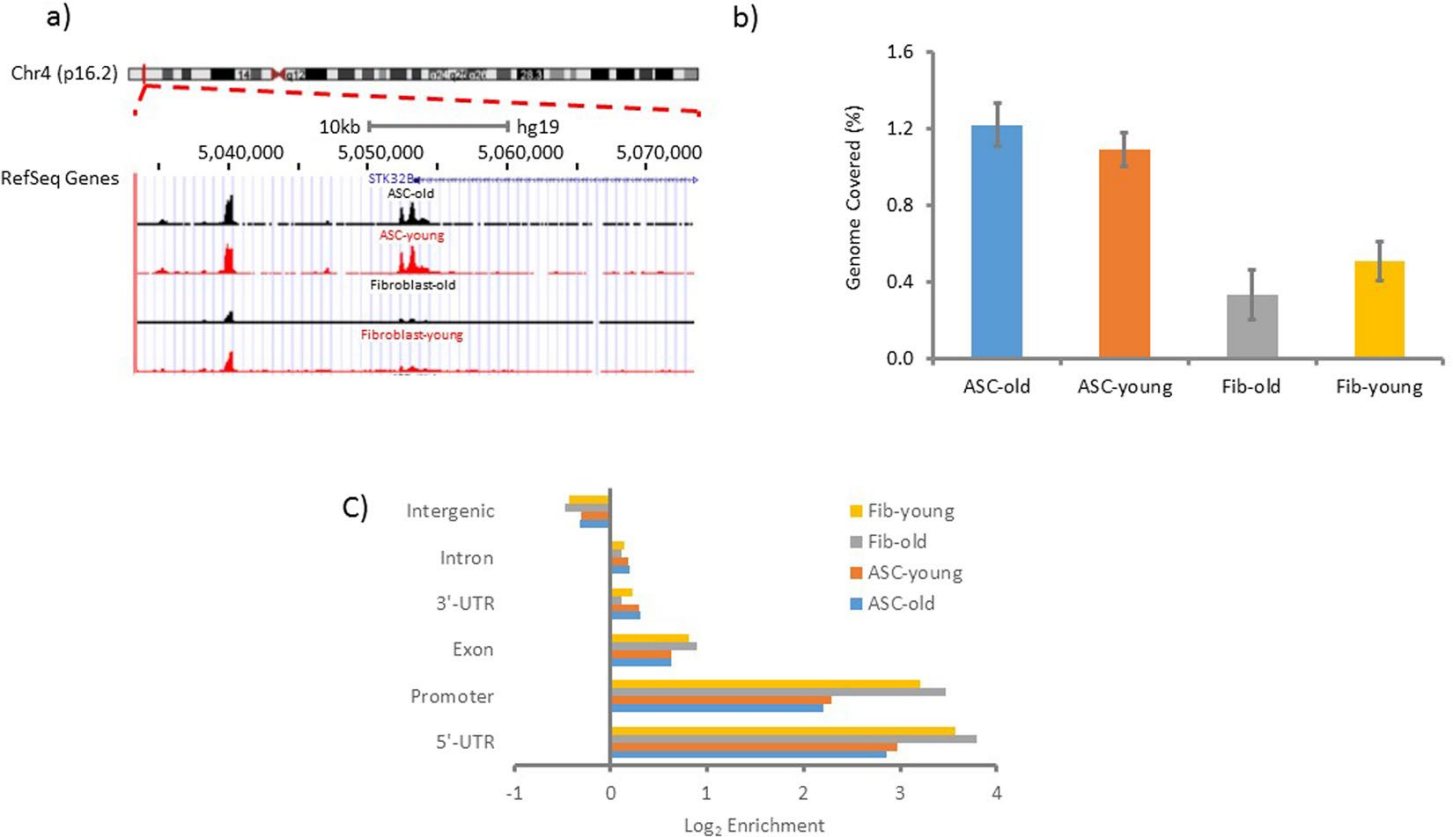
Genome coverage and enrichment of ATAC-seq peaks. Representative ATAC-seq peaks near TSS of *STK32B* on genome visualization tracks (**a**). For each cell type, an average track was generated by merging the individual tracks of all samples in the group. Tracks from top down are ASC-old, ASC-young, Fibroblast-old and Fibroblast-young. The average length of the genome covered by peaks in each sample group was normalized to the total length of the genome and presented as a percentage (**b**). The enrichment of peaks in the indicated genome regions was calculated using Homer software. Log_2_ enrichment was plotted for each sample group (**c**). n = 7 for ASC-old group; n = 6 for ASC-young group; n = 4 for fibroblast-old group and n = 4 for fibroblast-young group. Error bar denotes standard errors.

Using ATAC-seq technology, we examined differences in chromatin accessibility between ASCs and fibroblasts at baseline and during chronological aging. The premise of the ATAC-seq technology is that genome fragmentation by the Tn5 transposase preferentially targets open regions of chromatin. In this assay, the Tn5 transposome performs adaptor ligation and fragmentation simultaneously, and resulting fragments are identified by next-generation sequencing^28^. We investigated the Tn5 transposase accessibility profiles of primary human ASCs isolated from 6 young and 7 old donors and compared them with those of age-matched human fibroblasts from 4 young and 4 old donors (Table 1). The quantity of Tn5 transposase-generated DNA fragments is represented as peaks on genome visualization tracks, as exemplified in (Fig. 1a). The length of genome covered by the peaks was calculated for each sample group. As shown in (Fig. 1b), a higher percentage of the ASC genome is covered with peaks compared with that of fibroblast genome (1.22% for ASC-old, 1.09% for ASC-young, 0.33% for fibroblast-old and 0.51% for fibroblast-young), indicating that the chromatin is globally more accessible in ASCs across all ages. We then analyzed the enrichment of peaks in select annotated genome regions (Fig. 1c). In both ASCs and fibroblasts, accessible chromatin is highly enriched in the promoter and 5′-UTR regions (Fig. 1c). Interestingly, although the fibroblast genome is overall less accessible compared to the ASC genome, Tn5 transposase-generated peaks are relatively more enriched in the promoter and 5′-UTR regions in fibroblasts as compared to ASCs (9.63% for ASC-old, 10.23% for ASC-young, 23.31% for fibroblast-old and 15.76% for fibroblast-young). Thus, the result suggests that during fibroblast aging, promoter region becomes progressively more accessible contributing to associated aging phenotypes.

**Table 1:** Sample Features.

ASCs		Fibroblasts	
Age (Young Group)	Age (Old Group)	Age (Young Group)	Age (Old Group)
25	50	25	53
30	53	27	56
31	55	32	58
37	56	33	64
32	62		
35	63		
	75

**Figure 2:**
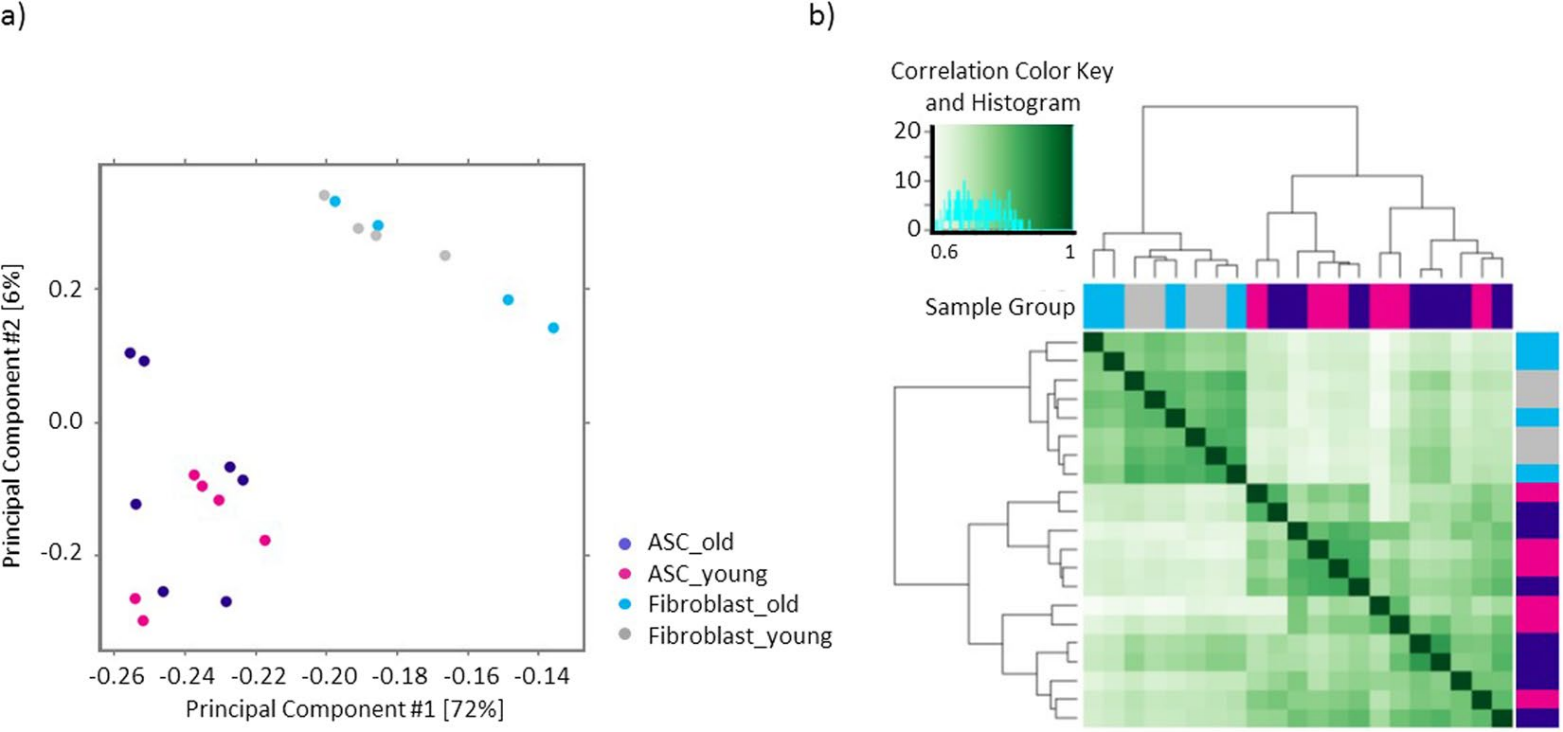
Sample correlation analysis based on ATAC-seq peak location and intensity. Principle component analysis (PCA) result of all samples is plotted as a 2D graph with principle component 1 as X-axis and principle component 2 as Y-axis (**a**). Similarity matrix analysis is shown as a heat map and the intensity of the color indicates cross-correlation between the two compared groups (**b**).

To further examine the global patterns of chromatin accessibility profiles of young and old ASCs and fibroblasts, we carried out principle component analyses (PCA) and similarity matrix analyses (Fig. 2), taking into consideration both peak location and intensity. PCA clearly differentiated ASCs and fibroblasts along principle component (PC)1 and PC2 axes, but the age difference was not clearly resolved by either PC1 or PC2, in either cell type (Fig. 2a). The correlation heatmap that was generated by the cross-correlation of every two samples based on their read counts in all merged peaks demonstrated a similar pattern. As shown (Fig. 2b), ASCs and fibroblasts are differentiated by well-separated clusters, however, within each cell type, no clear age-related clustering was observed, suggesting, not surprisingly, that age-dependent differences in patterns of Tn5 fragmentation are more subtle than those of cell-specific differences. These results are consistent with our prior transcriptional data and underscore the importance of deeper and more rigorous analyses, specifically at the promoter transcription start sites (TSS) regions during aging, in order to identify subtle, but important, age- and cell-specific epigenetic regulatory mechanisms.

### Genes with low transcriptional activity are characterized by less accessible TSS flanking regions

**Figure 3:**
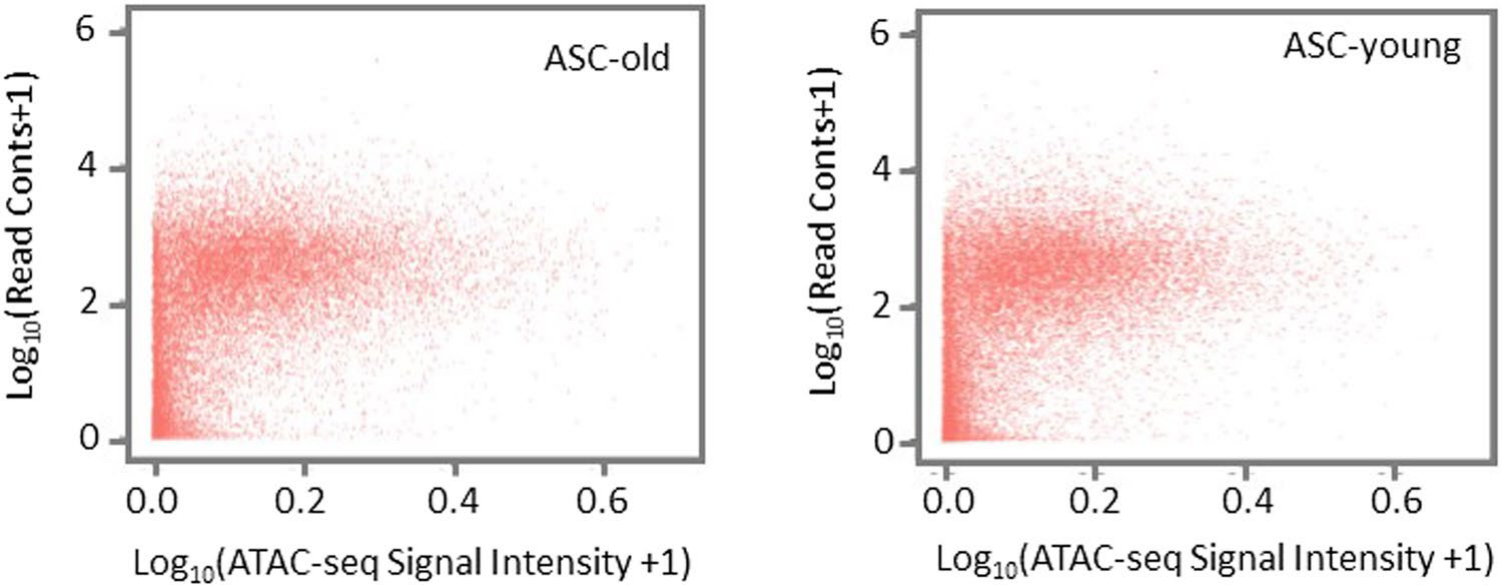
Correlation of gene expression level with promoter accessibility. Gene expression data was obtained from our previous transcriptome analysis^8^. Log_10_ transformation was performed for gene expression (RNA-seq read count) and promoter accessibility (ATAC-seq signal intensity in the promoter region) for ASC-old and ASC-young groups.

To assess the impact of chromatin accessibility in TSS flanking regions on gene expression in adult stem cells, we compared ASC ATAC-seq signal intensity in TSS flanking regions to transcript read counts from our ASC RNA-seq data set^8^. As shown in Fig. 3, genes with low transcript read counts tend to have less accessible TSS flanking regions, a feature that is maintained during ASC aging. Interestingly, genes expressed at overall higher levels demonstrate more variable accessibility profiles in TSS flanking regions, suggesting that chromatin accessibility may not be the primary determining factor for high transcript expression in ASCs and/or that chromatin accessibility in ASCs may a be more dynamic process than previously believed.

### Nucleosome position in flanking TSSs is globally well maintained during aging in ASCs but not in age-matched fibroblasts

**Figure 4:**
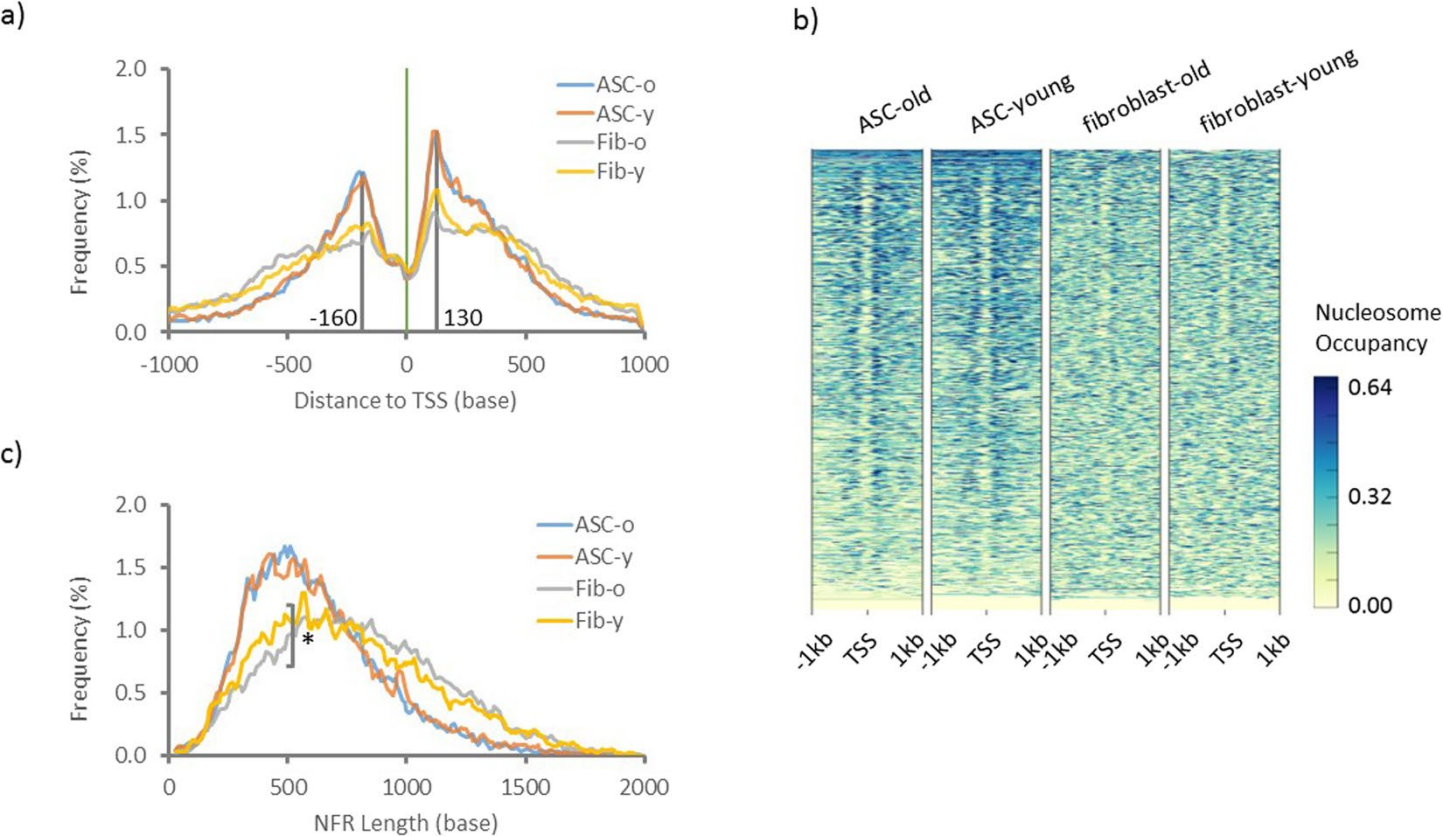
Distribution of normalized group average of +1 and −1 nucleosomes and lengths of NFR. Nucleosome positions were predicted by NucleoATAC software. The normalized distribution of each group average of +1 and −1 nucleosomes was plotted as a 3 points rolling average in (**a**). Nucleosome occupancy within +/− 1 kb of all TSS in mappable regions are shown as a heat map for all groups (**b**). The average distance between the +1 and −1 nucleosome pairs was calculated for each group and normalized before plotted as a 3 points rolling average in c). *Significant difference between the distributions, Kolmogorov-Smirnov test (α = 0.05).

To examine the impact of aging on chromatin structure on a finer level, we mapped the genome-wide nucleosome positions in young and old ASCs and age-matched fibroblasts via NucleoATAC^29^, a nucleosome position analysis software. Because nucleosomes flanking TSS, defined as −1 for up-stream of TSS and +1 for down-stream of TSS, have been implicated in the regulation of gene expression^16^, we specifically targeted these regions for the NucleoATAC analysis. We observed a distinctive difference between ASC and fibroblast young and old groups in the +1 and −1 nucleosome positions in promoter regions, indicating that aging has a differential impact on the two cell types. As shown in (Fig. 4a), the position of both +1 and −1 nucleosomes are globally well maintained during ASC aging, with the distribution of the +1 nucleosome being centered around +130, and the −1 nucleosome around −160 in both young and old samples. Fibroblasts, however, demonstrate a much broader distribution of nucleosomes than ASCs in young samples, with a noticeable expansion upon aging, suggesting that the position of nucleosomes in differentiated cells is not only more variable at baseline, but also, that this variability increases with aging in differentiated cells as compared to stem cells. Promoter region nucleosome occupancy patterns, as defined by nucleosomes located from −1kb to +1 kb from TSS, were similarly examined and are presented as heat maps in (Fig. 4b). These patterns consistently demonstrate that ASCs possess a tighter nucleosome position that is well maintained during aging, while fibroblasts possess broader and more variable nucleosome positions flanking the TSS across different ages.

We subsequently calculated the distance between pairs of +1 and −1 nucleosomes to investigate the length of the nucleosome free region (NFR) among all groups (Fig. 4c). Consistent with the aforementioned data, the lengths of the NFRs in ASCs reside most frequently around 500 bp range, a pattern that is specifically maintained during ASC aging. While the NFR length distribution is globally similar during aging in ASCs, in fibroblasts, the NFR length distribution widens with aging (Fig. 4c). Together, the data demonstrate that both the positioning of +1 and −1 nucleosomes as well as the distance between the pairs of nucleosomes, are well maintained during aging in adult stem cells. In contrast, noticeable changes of these chromatin features, especially a significant change of NFR length distribution (Kolmogorov-Smirnov test, α = 0.05), are observed during aging of differentiated cells. These results suggest that the chromatin structure in stem cells is globally more stable than that of somatic cells over time, and further, that gene transcription is likely to be altered via chromatin structure modifications during aging in somatic cells.

### Aging differentially impacts the +1 and −1 nucleosome positioning of genes specifically involved in protein sumoylation in both ASCs and fibroblasts

**Figure 5:**
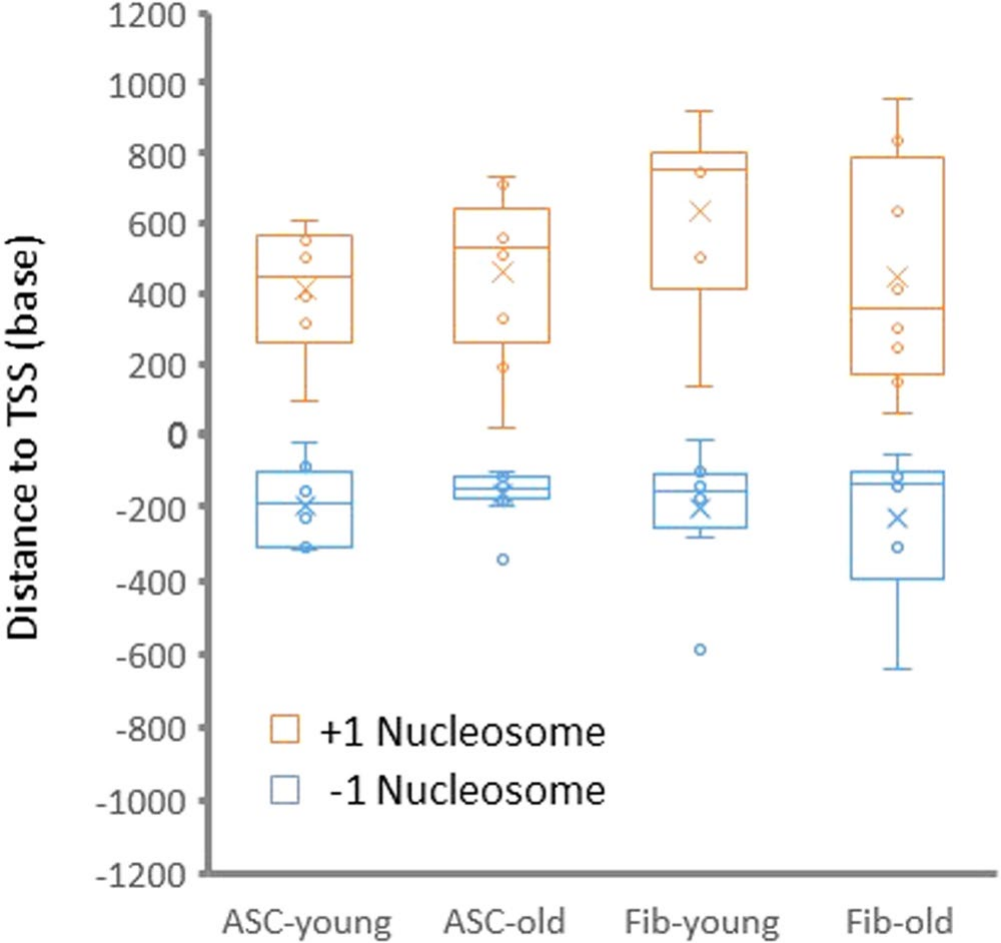
Distribution of nucleosomes flanking TSS of SUMO genes. The group average of +1 and −1 nucleosome position in the promoter regions of SUMO genes are shown as circles in a box plot. Orange color denotes +1 nucleosomes and blue −1 nucleosomes. Group means are denoted by x.

Although the +1 and −1 nucleosome position is globally well maintained in ASCs, we refined our search to identify for age-related changes at the individual gene level. To investigate specific pathways that are associated with changes in the positioning of the +1 and −1 nucleosomes during aging, we grouped genes by their age-related re-positioning of +1 and −1 nucleosomes, in both ASCs and fibroblasts. The enrichment of pathways in each group was analyzed using Reactome Pathway Database^30,31^. Using this approach, we demonstrated that, in fibroblasts, members of the protein sumoylation pathway are enriched in the group with minimal changes in the −1 nucleosome position between the young and old patients (Supplemental Table 1). In contrast, in ASCs, members of the sumoylation pathway were enriched in the group with minimal changes in the +1 nucleosome position between the young and old patients (Supplemental Table 2). To more specifically address these observations at the gene level, we compared the positions of the +1 and −1 nucleosomes of genes encoding Small Ubiquitin-like Modifier (SUMO) proteins. As shown in Fig. 5, the positioning of the +1 and −1 SUMO nucleosomes vary less during aging in ASCs than in fibroblasts, consistent with our initial observations of a global broadening of distribution of nucleosomes in aging fibroblasts (Fig. 5). Intriguingly, the interquartile range of the −1 nucleosome positions in ASCs from old patients were considerably less than that of young patients, while in fibroblasts, this trend was not observed. Since regions upstream of the TSS contain binding sites for factors involved in the control of gene transcription, these results suggest that the regulation of SUMO expression during aging may be governed, at least in part, by the alteration of chromatin structure, and further, that the regulatory mechanisms between fibroblasts and ASC may differ during aging in a cell-specific manner.

### SUMO-1 and SUMO-2,3 protein expression is stable in ASCs and fibroblasts during aging under non-stress conditions

**Figure 6:**
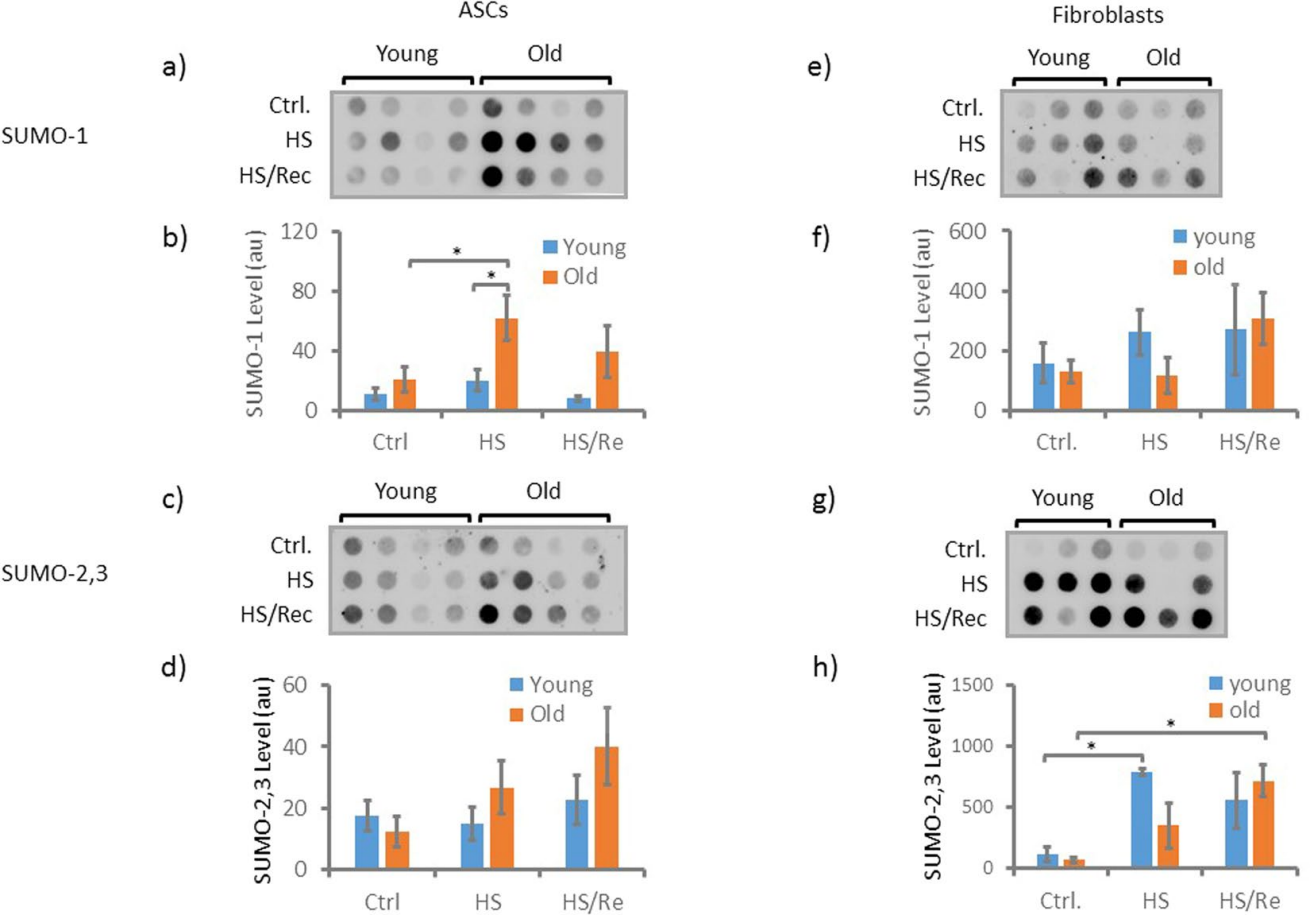
SUMO-1 and SUMO-2,3 protein expression. Dot blot analysis of global SUMO-1 expressions are shown in (**a**) for young and old ASCs, and (**e**) for young and old fibroblasts. Treatment conditions were: ctrl – at baseline without treatment; HS -heat shock at 42 °C for 1 h and HS/Rec: heat shock at 42 °C for 1 h followed by 1 h recovery at 37 °C. The intensities of the dots were quantified using ImageJ. Average intensities of the groups are plotted in (**b**) for ASCs and (**f**) for fibroblasts. Global SUMO-2,3 expressions are shown in (**c**) for young and old ASCs, and (**g**) for young and old fibroblasts. Average intensities of the groups are plotted in (**d**) for ASCs and in (**h**) for fibroblasts. Blue color denotes samples from young donors and orange old donors. n = 4 for each ASC group and n = 3 for each fibroblast group. Error bars indicate standard errors. *t-test, 2-tailed, p < 0.05.

Protein sumoylation, is a post-translational modification that affects protein structure and cellular localization, and is involved in diverse biological processes. To further investigate the impact of aging on protein sumoylation in ASCs and fibroblasts, we analyzed global SUMO-1 and SUMO-2,3 expression in young and old ASCs and fibroblasts using a protein dot blot assay. We observed no statistically significant differences in SUMO-1 and SUMO-2,3 protein quantities between cell type, irrespective of age, under non-stress conditions (Fig. 6, Ctrl). Of note, these results are concurrent with our previously published RNA-seq data^8^, demonstrating that the ratios of SUMO transcript levels in old vs. young are within 1.2 fold in both cell types (Supplemental Table 3). Together, the results indicate that under non-stress conditions, SUMO protein expression is not significantly altered during aging, despite cell-specific age-related positioning of +1 and −1 nucleosomes.

### SUMO-1 and SUMO-2,3 expression differs between ASCs and fibroblasts subject to heat shock-induced stress in a cell-specific manner during aging

Because aging has been specifically demonstrated to reduce organismal capacity in managing stress elicited by environmental stimuli, including heat shock^21^, and protein sumoylation is intimately involved in cellular responses to stress^23,27^, we investigated the response of SUMO protein expression to heat shock-induced stress in ASCs and fibroblasts between young and old sample groups. We examined global SUMO protein expression levels in ASCs and fibroblasts, both before and after heat shock (HS), as well as after recovery from HS. We observed that after 1 h of HS at 42 °C, the SUMO-1 protein level was significantly (p < 0.05) increased in ASC-old as compared to prior to HS (Fig. 6a,b). Further, after 1 h of HS, ASC-old demonstrated significantly (p < 0.05) higher levels of SUMO-1 compared to ASC-young samples subject to HS (Fig. 6a,b). The 1 h recovery period did not lead to a statistically significant decrease of SUMO-1 levels in ASC-old (Fig. 6a,b). The ASC-young samples demonstrated no statistically significant changes in SUMO-1 levels between the ctrl, HS and HS recovery conditions (Fig. 6a,b). SUMO-2,3 levels in ASC-old samples followed a similar trend to that of SUMO-1, with increased levels after HS and recovery conditions, however, the trend was not statistically significant between conditions or when compared to the ASC-young group (Fig. 6c,d). The ASC-young samples again demonstrated no statistically significant changes in SUMO-2,3 levels between the ctrl, HS and HS recovery conditions, similar to SUMO-1.

Fibroblasts demonstrated strikingly different SUMO protein levels from ASCs after HS and recovery, in an age-related manner. Specifically, SUMO-2,3 levels were significantly (p < 0.05) increased in the fibroblast-young group after HS, an increase that was slightly reduced after the 1 h recovery period (Fig. 6g,h). The fibroblast-old group similarly demonstrated increasing SUMO-2,3 levels after HS, an augmentation that became significant after recovery from HS (Fig. 6g,h). A similar pattern demonstrating increased SUMO-1 levels after HS and recovery were observed for fibroblast-young and fibroblast-old groups, respectively, although no statistically significant changes were detected (Fig. 6e,f).

**Figure 7:**
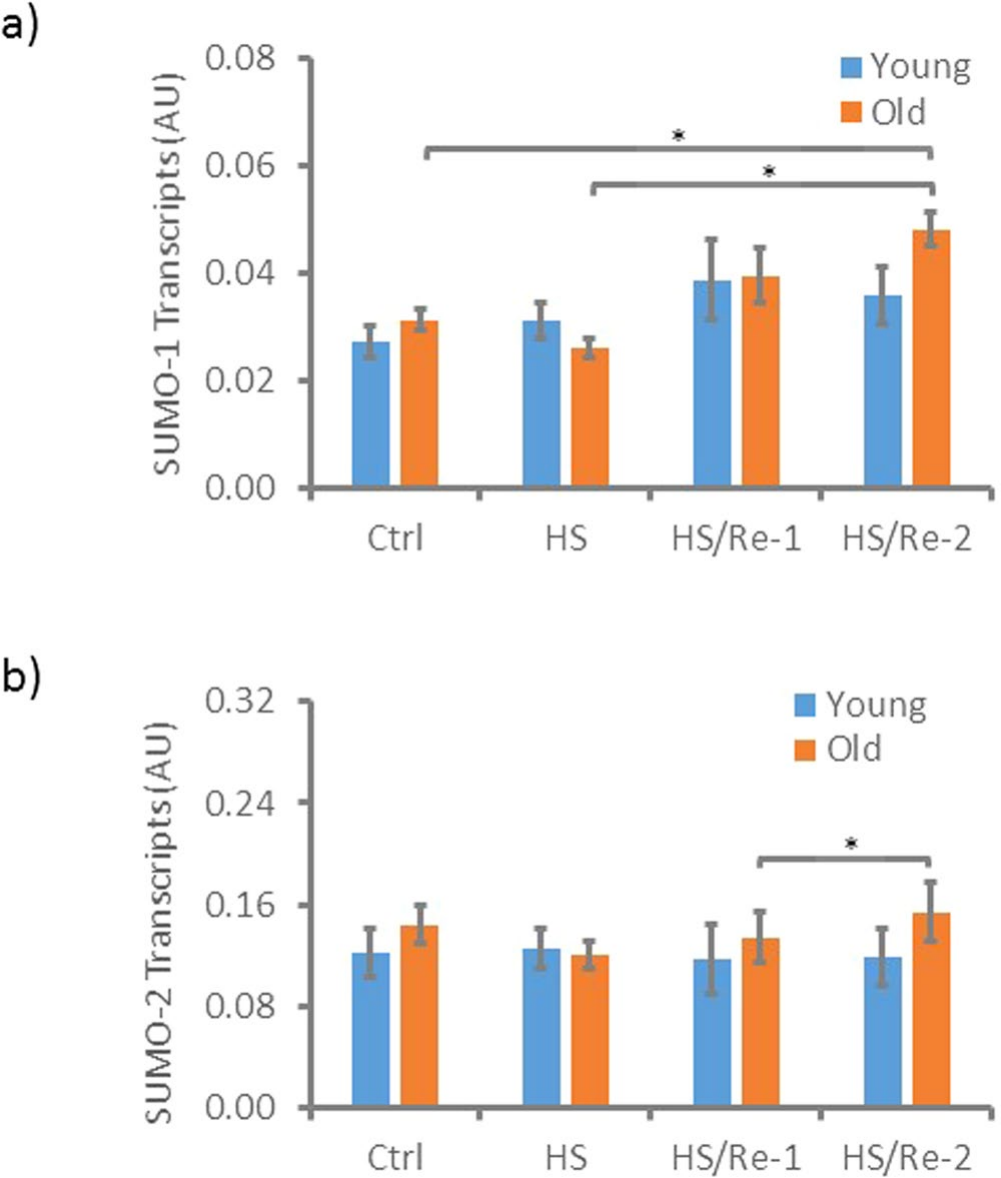
SUMO1 and SUMO2 gene transcription. Expression levels of SUMO1 and SUMO2 in ASCs under stress and non-stress conditions were determined by real-time PCR analysis. SUMO1 and SUMO2 levels were normalized to that of GAPDH and shown in (**a**,**b**) respectively. Ctrl: without treatment; HS: heat shock at 42 °C for 1 h; HS/Re-1: heat shock at 42 °C for 1 h followed by 1 h recovery at 37 °C; HS/Re-2: heat shock at 42 °C for 1 h followed by 2 h recovery at 37 °C n = 3. Error bars denote standard errors. *t-test, 2-tailed, p < 0.05.

Gene expression is regulated at multiple levels, including the rate of transcription, RNA processing, mRNA stability, and protein translation. We measured age-related changes in the transcript levels of SUMO-1 and SUMO-2 in ASCs in response to HS and recovery from HS. We detected a statistically significant increase of SUMO-1 transcript levels in ASCs from old, but not young, patients after a 2-hour recovery following HS (Fig. 7a). Changes of SUMO-2 transcript levels are more subtle compared to SUMO-1. A statistically significant increase in SUMO-2 was observed in the ASCs from old donors after a 2-hour recovery compared to 1-hour recovery after HS. These data are consistent with our protein expression data and indicate that the observed changes in SUMO protein expression in ASCs subject to HS is at least partly regulated at the transcriptional level.

Together, our data suggest that ASCs and fibroblasts respond differently to HS-induced stress and recovery via distinct SUMO protein level alterations in a cell-specific manner during aging. Aging ASCs demonstrate overall increased SUMO protein level expression after stress exposure, a pattern that is significant for SUMO-1. Surprisingly, in contrast, young ASCs demonstrate minimal shifts in SUMO protein levels after HS or recovery. In contradistinction, young fibroblasts demonstrate a more robust increase in global SUMO expression relative to old fibroblasts under these conditions. While both SUMO-1 and SUMO-2,3 expression are increased post HS and recovery in young and old fibroblasts, this pattern is statistically significant only for the SUMO-2,3 protein, in contrast to the significant increase in SUMO-1 after stress in old ASCs.

These data are concurrent with our observed differential, cell-specific, age-related shifts in positioning of +1 and −1 nucleosomes in the promoter regions of SUMO genes and support an important role for alterations in chromatin structure as a cell- and age-specific mechanism for the functional regulation of protein sumoylation in response to cellular stress.

## Discussion

In this study, we employed ATAC-seq to examine the chromatin accessibility profiles of human ASCs and fibroblasts in a cell-specific manner during aging. Our data demonstrates distinctive differences in Tn5 digestion patterns between the two cell types, with ASCs displaying a globally more open chromatin structure that is well maintained during aging. The distribution patterns of +1 and −1 nucleosome position further reveal a differential impact of aging on ASCs and fibroblasts. ASCs are characterized by well-maintained nucleosome positions during aging, in contrast to fibroblasts, whose nucleosome positions are altered during aging. More detailed examination of differential positioning of +1 and −1 nucleosomes, specifically in promoter region, led to identification of differential nucleosome positions in the promoters of genes involved in the protein sumoylation pathway in both cell types. To determine whether there is a regulatory link between differential nucleosome positioning and gene expression, we investigated SUMO protein expression under non-stress and stress conditions in ASCs and fibroblasts in relation to aging. As suggested by differential chromatin accessibility profiles, we observed a concurrent differential expression of SUMO proteins in an age- and cell-specific manner in response to heat shock. Together our data reveal that subtle changes in nucleosome positions during aging is correlated with differential stress response in ASCs, suggesting that nucleosome position change is a distinct mechanism regulating the stress response in human adult stem cells.

In both stem and differentiated cells, Tn5 fragmentation is highly enriched in promoter regions compared to other regions. Previous studies have associated chromatin accessibility in the promoter region with regulation of gene expression^16^. As such, we compared the promoter chromatin accessibility profiles relative to their transcriptional activity, as examined in our prior genome-wide transcriptional profiling of ASCs and fibroblasts^8^. As expected, our data demonstrate that in ASCs, the genes expressed at low levels tend to have less accessible promoters. However, genes expressed at high levels have a wide variability in their promoter accessibility profiles. This is consistent with prior studies in other organisms and cells^17–19^. Thus, genes expressed at high levels are characterized by variably open chromatin in promoter regions, suggesting that differential binding of transcription factors play an important role in regulating expression of these genes.

In prior studies, the nucleosome position flanking the TSS has been shown to influence gene expression in both yeast and human CD4^+^ T cells^32–34^. Considering our observations that the nucleosome position profiles of ASCs and fibroblasts are distinctively different, and more importantly, that the promoter nucleosome profiles of ASCs are globally well maintained during aging while that of the fibroblasts are subject to wide variability during aging, our results further enforce the link between specific chromatin conformations and aging in a cell-specific manner. These results are consistent with the changes in the age-specific transcriptomes of ASCs and fibroblasts that we previously reported. Consequently, we believe that the stability of nucleosome position profiles and transcriptome activity are tightly linked in ASCs, reflecting the critical homeostatic mechanisms intrinsic to stem cells, in contrast to differentiated cells^35,36^, such fibroblasts.

Although the nucleosome positioning profiles of ASCs are globally stable during aging, we successfully identified age-related changes in the nucleosome position of specific regulatory pathways including protein sumoylation, a post-translational modification. Protein sumoylation is increasingly shown to be implicated in many cellular processes, including gene expression regulation and maintenance of chromatin integrity^37^. Sumoylation has been shown to regulate the function of proteins by specifically modifying their stability, protein-protein interactions, and cellular localization^37^. The mammalian SUMO protein family is composed of four members, SUMO-1-4, with SUMO-4 being the least understood^38^. SUMO-1 is ~50% homologous with SUMO-2 and SUMO-3, while SUMO-2 and SUMO-3 are 97% homologous with one another^39^. Although protein sumoylation by the SUMO variants affect cellular processes differently, genetic and biochemical studies show that the 3 variants may play partially redundant roles^40–42^. *In vivo*, SUMO-1 and SUMO-2,3 conjugate to distinct substrates and their ability to form polymeric SUMO chains differ^39^. SUMO-2 and SUMO-3 contain internal sumoylation sites that link SUMO molecules. SUMO-1 can be incorporated in these chains but the lack of internal consensus sumoylation sites in SUMO-1 hinders further chain elongation^43^. In addition, SUMO-1 prefers to be associated with the nuclear envelope and nucleolus, while SUMO-2,3 are localized mainly in the nucleoplasm^44^. The SUMO-4 mRNA appears to be expressed only in kidney and spleen cells^45^, however, *in vivo* expression of SUMO-4 has yet to be reported^38^.

Our investigation of SUMO1-3 in human ASCs and fibroblasts reveal that under non-stress conditions, SUMO1-3 protein expression is stable during aging under non-stress conditions. However, in response to heat shock, SUMO-1 is significantly upregulated in old ASCs, but not in old fibroblasts. Because SUMO-1 and SUMO-2,3 differ in their binding sequence and subcellular localization, their roles in cellular stress response are likely to differ. Our data point to a specific role for SUMO-1 in regulating the stress response during aging in primary human adult stem cells. SUMO-2,3 protein expression after heat shock are only significantly increased in young fibroblasts, suggesting an age-dependent dysregulation of this pathway in aged differentiated cells. Our data demonstrates that the promoter nucleosome position of SUMO genes in old fibroblasts becomes more variable as compared to young fibroblasts. In contrast, the −1 nucleosome position in old ASCs resides within a narrower range. Although the promoter nucleosome position is not the single determining factor for transcriptional activation, a permissive positioning of nucleosomes could allow transcription to proceed when necessary transcriptional factors become available. It is thus possible that the difference in nucleosome positioning in aging ASCs and fibroblasts results in differential SUMO expression under stress conditions.

Since adult human stem cells are critical for tissue homeostasis and regeneration, they must be equipped with robust protective mechanisms against a variety of intrinsic and extrinsic insults. We hypothesize, based on our data, that the increased sensitivity of SUMO expression in old ASCs in response to heat shock may represent a stem cell-specific protective regulatory mechanism against the accumulation of age-dependent defects arising from intrinsic or extrinsic cellular stress. In support of this hypothesis, SUMO-1 has been shown to be an important modulator of the heat shock transcription factor 1 (*HSF1*) function in response to stress in HeLa cells^46^. Further, SUMO-2,3 protein expression is required for human bone osteosarcoma epithelial cells to survive heat shock in^27^. In fact, at least 120 SUMO substrates involved in a diverse set of cellular processes have been identified in mammals, suggesting a broad and important role for these proteins in multiple cellular pathways^37^.

In summary, our data demonstrate that the global chromatin structure in human ASCs is more accessible and stable than the chromatin structure in fibroblasts. Further, the promoter nucleosome position profile in ASCs is distinctively different from fibroblasts, a feature that is also stable during aging in ASCs, but not in fibroblasts. Subtle changes in the nucleosome position in the promoter regions of genes encoding SUMO proteins reveal a narrower distribution of the −1 nucleosomes in old ASCs, as compared to a more variable distribution in old fibroblasts. These observations correlate with increased upregulation of SUMO expression in response to heat shock-induced stress in old ASCs, in contradistinction to old fibroblasts. These findings provide important new insights into the understanding of chromatin structure, transcriptional regulation, and post-translational modification during aging in human stem and differentiated cells.

## Methods

### Tissue procurement

The study was approved by the Institutional Review Board of University of Pennsylvania (IRB approval Protocol number 812150). Informed consent was obtained from all tissue donors and all methods were performed in accordance with the relevant guidelines and regulations. Subcutaneous abdominal adipose tissues were excised from healthy donors during abdominoplasties. The tissues were immediately transferred to the laboratory. The adipose tissue was dissected from skin and stored at −70 °C in 50 mL conical tubes until ASC isolation. No cryopreservation or other agents were used in the freezing of the adipose tissue specimens. Skin specimens were processed immediately for dermal fibroblasts without storage at −70 °C.

### Isolation and *in vitro* culturing of ASCs and fibroblasts

ASCs were isolated from 13 tissue samples of females ranging in age from 25 to 75, according to a standard collagenase protocol^47^. Isolated ASCs were cultured in Dulbecco’s Modified Eagle Medium/F12 (Gibco Life Technologies Co., Norwalk, CT) supplemented with 100 I.U./mL penicillin and 100 (μg/mL) streptomycin (Gibco Life Technologies Co.) and 10% FBS (Serum Source International, Charlotte, NC) at 37 °C with 5% CO_2_. The culture media was changed every three days. All analyses were conducted with early passage ASCs (p < 4).

Fibroblasts were isolated from 8 females ranging in age from 25 to 64, using the method previously described^48^. The cells were cultured under 5% CO_2_ at 37 °C, and the culture media, same as that used for ASCs, were changed every three days. All analyses were conducted with early passage fibroblasts (p < 7).

### ATAC-seq sample preparation

Approximately 150,000 ASCs or fibroblasts isolated from each tissue specimen were used to prepare ATAC-seq libraries according to published protocols^28,49^. The quality of libraries was assessed using a Bioanalyzer HS-DNA chip (Agilent). Concentrations were quantified using the KAPA Library Quantification Kit (Sigma-Alderich). Pooled, barcoded ATAC-seq libraries were sequenced on the Illumina NextSeq. 500 platform to generate 75-bp paired-end reads.

### ATAC-seq data processing

Sequencing reads were trimmed using Trimmomatic^50^ to remove Illumina adapters and low quality base calls on both ends (Phred < 3). Trimmed reads were mapped to the reference human genome assembly GRCh37 (hg19) using Bowtie2^51^, allowing fragments up to 2 kb to be aligned (“-X 2000”). The alignment result was further processed by SAMtools^52^ to remove low quality alignments (mapping quality >10), duplicate reads, reads mapped to mitochondria, and reads mapped to ENCODE blacklisted genomic regions. All mapped reads were offset by +4 bp for the +strand and −5 bp for the -strand. The distribution of paired-end sequencing fragment sizes was assessed using Qualimap^53^.

### Track visualization, peak calling and differential analysis

Genome-wide tag density was computed using BEDtools^54^ and UCSC command line utilities to generate tracks for visualization, for which purpose the visualization tracks were normalized to one million reads per sample. For each cell type, an average track was generated by merging replicate tracks using BEDtools (“unionbedg”) and using the average tag density of replicate tracks as the final tag density. MACS2^55^ was used to identify open chromatin regions (or peaks) for each replicate, requiring peak calling FDR to be lower than 0.05 (with parameter “-q 0.05 -nomodel -shift 37 -extsize 73”). Peaks were merged for the same cell types using BEDtools. Individual peaks separated by <100 bp were stitched together. Gene target for each peak was identified as the gene with nearest TSS to the peak, using HOMER suite (“annotatePeaks -size given”)^56^. The HOMER annotation process also performed compartment analysis and enrichment analysis after separating the peaks into four categories: Intergenic, Intron, Promoter-TSS (−1kb to +100 bp of TSS) and Others.

### Nucleosome positioning analysis

Nucleosome positions were calculated using the NucleoATAC software^29^ package with default parameters. RefSeq TSS regions (−1 kb to 1 kb around each TSS) were used as input regions of interest for NucleoATAC analysis. Position of the immediate upstream nucleosome (−1) and downstream nucleosome (+1) for each TSS were used for downstream analysis.

### Pathway enrichment analysis

Genes were grouped according to the change of positioning of +1 and −1 nucleosomes during aging, including <30 bases and >30 bases for either or both promoter nucleosomes. Previous studies considered the dyads of nucleosomes that differ by 10–20 as “well-localized”^16^. We used 30 bp to take into the account the higher innate variability of human samples. The grouped genes were analyzed for pathway enrichment using Reactome Pathway Database^30,57^.

### Heat shock treatment of ASCs and fibroblasts

ASCs and fibroblasts were seeded in 12 well plates and cultured to 80% confluence at 37 °C under 5% CO_2_. For heat shock, the cells were transferred to an incubator maintained at 42 °C with 5% CO_2_ and incubated for 1 h. At the end of the incubation, cells were briefly washed with PBS and proteins were extracted on plate using RIPA lysis buffer (VWR) with protease inhibitors (Roche Life Science) at 4 °C for 30 min. For heat shock recovery experiments, the cells were incubated at 37 °C under 5% CO_2_ for 1 h after heat shock. Cell lysates were isolated as described above. Whole cell lysate from untreated cells were processed using the same procedure. The cell lysates were cleared of debris by centrifugation at 4 °C for 15 min. Protein concentration was quantified using the BCA assay (Thermo Fisher Scientific, MA) according to the manufacturer’s instructions.

### Global SUMO expression analysis by protein dot blot

Dot blot analysis was carried out as described by Castoralova *et al*.^58^. Briefly, 20 μg of cell lysates were diluted to a final volume of 200 μL with PBS (Life Technologies) and loaded onto a dot-blot device, Minifold-I 96-Well System (GE Healthcare Life Sciences), following the manufacturer’s instructions. The primary antibodies used were: rabbit anti-human SUMO-1 and SUMO-2,3 (Cell Signaling Technology, Danvers, MA). The secondary antibody used was HRP conjugated anti-rabbit IgG (Cell Signaling Technology). The blots were developed using ECL™ Western Blotting Detection Reagents (GE Healthcare Bio-Sciences). Protein dots were visualized using Amersham Imager 600 (GE Healthcare Bio-Sciences). Intensity of each dot was analyzed using Image J^59^.

### Real-time quantitative PCR analysis of gene transcription

Total RNA was isolated using TRIzol reagent (Invitrogen) from control and HS treated ASCs of 3 young and 3 old donors and cDNA was synthesized with oligo(dT) primers from 0.5 μg of total RNA using the High-Capacity RNA-to-cDNA kit (Applied Biosystems) according to the manufacturer’s instructions. PCR amplification was carried out using SYBR™ Green PCR Master Mix (Applied Biosystems) on 7900HT Fast Real-Time PCR System (Applied Biosystems). Cycling conditions included pre-denaturation at 95 °C for 10 min followed by 40 cycles of denaturation at 95 °C for 15 s and extension at 60 °C for 1 min. All primers are listed in Supplemental Table 4. LinRegPCR software was used for data analysis^60^ and SUMO transcript levels were normalized to that of GAPDH.

### Statistical analysis

For data analyses, two-tailed Student’s t-tests and Kolmogorov-Smirnov test^61,62^ were performed using Excel 2016. In all tests, values of P < 0.05 were considered statistically significant.

### Data availability statement

The datasets supporting the conclusions of this article are available in the standard public repository GEO (the Gene Expression Omnibus, https://www.ncbi.nlm.nih.gov/geo/query/acc.cgi?acc=GSE105077).

## Electronic supplementary material

Supplemental Information
