# Supplementary material for: Age Alters Chromatin Structure and Expression of SUMO Proteins under Stress Conditions in Human Adipose-Derived Stem Cells
Source: Sci Rep. 2018 Jul 31;8:11502. doi: 10.1038/s41598-018-29775-y (PMC6068198; doi:10.1038/s41598-018-29775-y)

Age Alters Chromatin Structure and Expression of SUMO Proteins under Stress Conditions in Human Adipose-Derived Stem Cells

Xiaoyin Shan[1], Cleresa Roberts[1], Yemin Lan[2], Ivona Percec[1*]

[1]Department of Surgery, University of Pennsylvania, Philadelphia, PA 19104, USA
[2]Epigenetics Institute, Department of Cell and Developmental Biology, University of Pennsylvania, Philadelphia, PA 19104, USA

* Correspondence:

Ivona Percec, Department of Surgery, Perelman School of Medicine, University of Pennsylvania, 9-135 Smilow Center for Translational Research, 3400 Civic Center Blvd, Philadelphia, PA 19104 USA. Tel: (215) 662-7300, Fax: 215-349-5895, email: ivona.percec@uphs.upenn.edu

Supplemental Table 1. Enriched Pathways in Fibroblast Genes with -1 Nucleosome Show Minimal Changes in Positioning during Chronological Aging.

Pathway identifier	Pathway name	#Entities found	#Entities total	Found/Tota (%)	Entities pValue	Entities FD
R-HSA-2990846	SUMOylation	10	125	8.00	0.03	0.49
R-HSA-3065678	SUMO is transferred from E1 to E2 (UBE2I, UBC9)	3	7	42.86	0.00	0.49
R-HSA-3065679	SUMO is proteolytically processed	2	6	33.33	0.02	0.49
R-HSA-3065676	SUMO is conjugated to E1 (UBA2:SAE1)	2	8	25.00	0.04	0.49
R-HSA-3108232	UMO E3 ligases SUMOylate target proteins	9	116	7.76	0.04	0.49
R-HSA-4615885	SUMOylation of DNA replication proteins	5	48	10.42	0.04	0.49

Supplemental Table 2. Enriched Pathways in ASC Genes with +1 Nucleosome Show Minimal Changes in Positioning during Chronological Aging.

Pathway identifier	Pathway name	#Entities found	#Entities total	Found/Total (%)	Entities pValue	Entities FDR
RR-HSA-2990846	SUMOylation	15	125	12.00	0.03	0.73
R-HSA-3108232	SUMO E3 ligases SUMOylate target proteins	15	116	12.93	0.02	0.73
R-HSA-3232118	UMOylation of transcription factors	5	20	25.00	0.01	0.73
R-HSA-4615885	SUMOylation of DNA replication proteins	7	48	14.58	0.05	0.73

Supplemental Table 3. Ratios of Transcript Level in Old vs. Young ASCs and Fibroblasts. *

	ASC O/Y	Fib O/Y
SUMO1	1.1	1.0
SUMO2	1.0	1.1
SUMO3	1.2	1.1

* Transcripts levels were obtained from the data set discribed in Shan, X. et al. Transcriptional and Cell Cycle Alterations Mark Aging of Primary Human Adipose-Derived Stem Cells. *Stem Cells* **35**, 1392-1401 (2017).

Supplemental Table 4. Primers used for Real-time Quantitative PCR Analysis of Gene Transcription.

Gene Symbol	Forward Primer	Revers Primer
**SUMO1***	5’-CAGGAGGCAAAACCTTCAAC	5’-TCCATTCCCAGTTCTTTTGG
**SUMO2***	5’-GGATGGTTCTGTGGTGCAGT	5’-TTCCAACTGTGCAGGTGTGT
**GAPDH**	5’-AGAAGGCTGGGGCTCATTTG	5’-AGGGGCCATCCACAGTCTTC

*: Primers were previously reported in: Li, S. et al. Analysis of gene expression in single human oocytes and preimplantation embryos. *Biochem. Biophys. Res. Commun.* **340,** 48-53 (2006)

Supplemental Figure 1. Original dot blots for figure 6

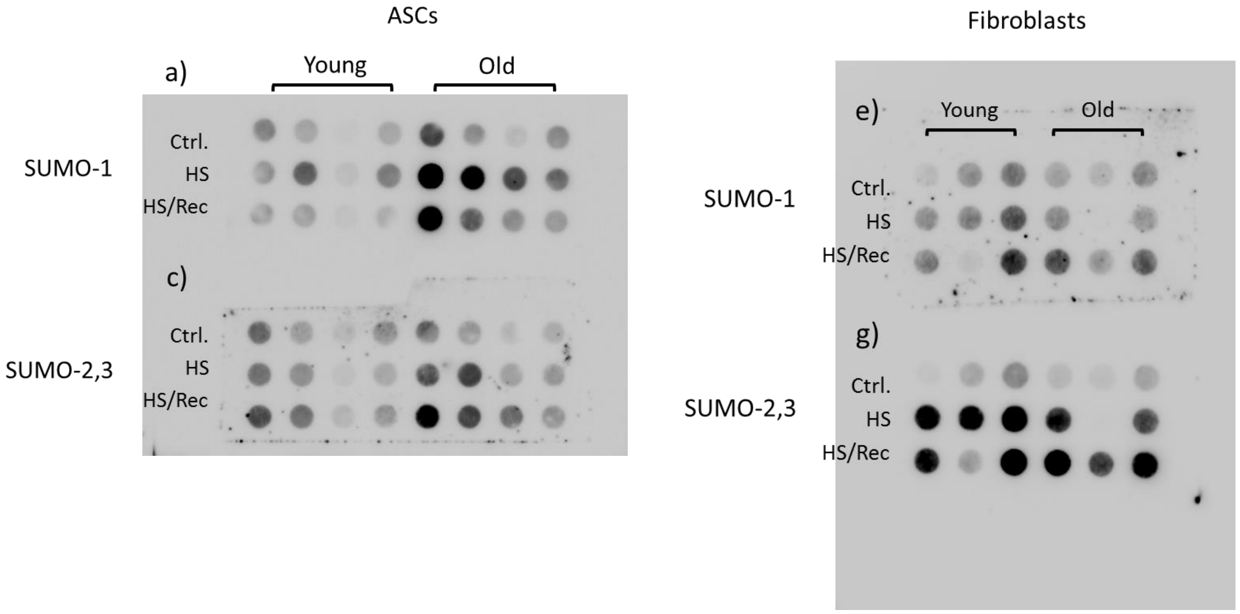

Supplement: Supplementary file 1 — Supplemental Information [file 41598_2018_29775_MOESM1_ESM.pdf]
